# Modeling Influenza Virus Infection: A Roadmap for Influenza Research

**DOI:** 10.3390/v7102875

**Published:** 2015-10-12

**Authors:** Alessandro Boianelli, Van Kinh Nguyen, Thomas Ebensen, Kai Schulze, Esther Wilk, Niharika Sharma, Sabine Stegemann-Koniszewski, Dunja Bruder, Franklin R. Toapanta, Carlos A. Guzmán, Michael Meyer-Hermann, Esteban A. Hernandez-Vargas

**Affiliations:** 1 Systems Medicine of Infectious Diseases, Department of Systems Immunology and Braunschweig Integrated Centre of Systems Biology, Helmholtz Centre for Infection Research, Braunschweig 38124, Germany; Alessandro.Boianelli@helmholtz-hzi.de (A.B.); nguyenkinh@ytecongcong.com (V.K.N.); 2 Department of Vaccinology and Applied Microbiology, Helmholtz Centre for Infection Research, Braunschweig 38124, Germany; Thomas.Ebensen@helmholtz-hzi.de (T.E.); Kai.Schulze@helmholtz-hzi.de (K.S.); CarlosAlberto.Guzman@helmholtz-hzi.de (C.A.G.); 3 Department of Infection Genetics, Helmholtz Centre for Infection Research, Braunschweig 38124, Germany; Esther.Wilk@helmholtz-hzi.de; 4 Immune Regulation, Helmholtz Centre for Infection Research, Braunschweig 38124, Germany; Niharika.Sharma@helmholtz-hzi.de (N.S.); Sabine.Stegemann-Koniszewski@helmholtz-hzi.de (S.S.-K.); Dunja.Bruder@helmholtz-hzi.de (D.B.); 5 Infection Immunology, Institute of Medical Microbiology, Infection Control and Prevention, Otto-von-Guericke-University, Magdeburg 39106, Germany; 6 Center for Vaccine Development, University of Maryland School of Medicine, Baltimore, Maryland 21201, USA; ftoapanta@medicine.umaryland.edu; 7 Department of Systems Immunology and Braunschweig Integrated Centre of Systems Biology, Helmholtz Centre for Infection Research, Braunschweig 38124, Germany; mmh@theoretical-biology.de; 8 Institute for Biochemistry, Biotechnology and Bioinformatics, Technische Universität Braunschweig, Braunschweig 38106, Germany

**Keywords:** mathematical models, parameters estimation, influenza, coinfection, aging, vaccinology, host genetic factors

## Abstract

Influenza A virus (IAV) infection represents a global threat causing seasonal outbreaks and pandemics. Additionally, secondary bacterial infections, caused mainly by *Streptococcus pneumoniae*, are one of the main complications and responsible for the enhanced morbidity and mortality associated with IAV infections. In spite of the significant advances in our knowledge of IAV infections, holistic comprehension of the interplay between IAV and the host immune response (IR) remains largely fragmented. During the last decade, mathematical modeling has been instrumental to explain and quantify IAV dynamics. In this paper, we review not only the state of the art of mathematical models of IAV infection but also the methodologies exploited for parameter estimation. We focus on the adaptive IR control of IAV infection and the possible mechanisms that could promote a secondary bacterial coinfection. To exemplify IAV dynamics and identifiability issues, a mathematical model to explain the interactions between adaptive IR and IAV infection is considered. Furthermore, in this paper we propose a roadmap for future influenza research. The development of a mathematical modeling framework with a secondary bacterial coinfection, immunosenescence, host genetic factors and responsiveness to vaccination will be pivotal to advance IAV infection understanding and treatment optimization.

## 1. Introduction

Seasonal and pandemic influenza A virus (IAV) infections remain major causes of severe morbidity and mortality worldwide. They cause enormous economic loss and serious adverse events leading to hospitalization, health complications, and death [1,2,3]. The outbreaks reported as the Asiatic Flu (H2N8) (1889–1890), Spanish Flu (H1N1) (1918–1920), Asian Flu (H2N2) (1957–1958), Hong Kong Flu (H3N2) (1968–1969) and more recently the swine Flu (H1N1) (2009) have revealed a high morbidity and mortality rate [4]. Particularly, IAV related diseases are more severe among high risk groups: toddlers (<2 years old), pregnant women, seniors (≥65 years old) and immunocompromised people [5]. Moreover, IAV infection is influenced by continuous antigenic drifts, antigenic shifts and host factors [6], requiring vaccination strategies to be reformulated continuously [7,8]. As a result, new severe epidemics and pandemics are likely to occur.

Advanced knowledge of the immune system during IAV infection is a key step towards the establishment of improved prevention, control and treatment strategies for IAV infection. To this end, in recent years mathematical modeling has risen as a promising tool to quantify IAV infection as well as to identify potential therapeutic targets to control IAV. Mathematical models have been developed with different grades of detail providing quantitative insights into viral dynamics within a host [9,10,11]. Although the picture of IAV kinetics resulting from mathematical modeling and experimental studies has improved, knowledge of the mechanisms of viral infection control, immune modulation, and bacterial coinfection is still largely fragmented. Three excellent reviews in influenza mathematical modeling can be found in [9,10,11]. In this paper, we update the state of the art of the mathematical models for IAV infection, focusing mainly on parameter estimation issues. To this end, we go beyond the review presenting a mathematical model to exemplify different problems in mathematical biology. In the last section, we highlight relevant open questions, creating a roadmap for mathematical modeling towards a global understanding of influenza infection research.

### IAV Pathogenesis

**Figure 1:**
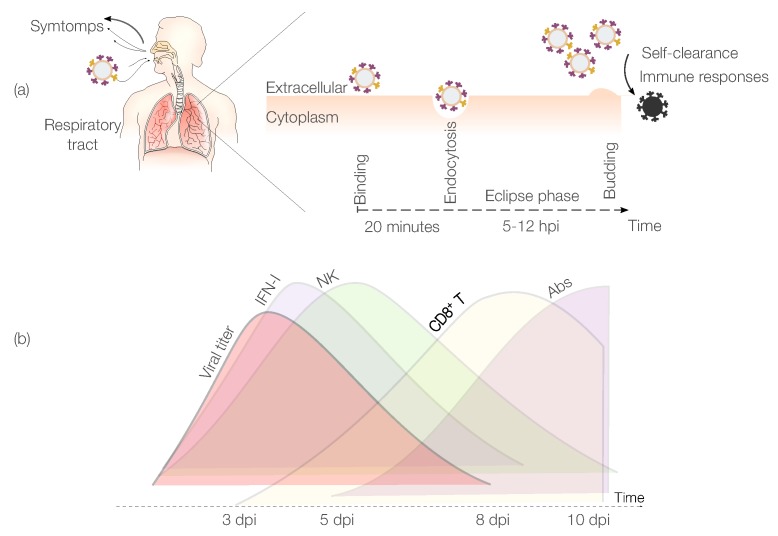
Influenza A virus (IAV) infection and dynamics. (**a**) Description of the main phases of IAV infection within a host. After entering the respiratory tract, each virion binds to a target cell. Then, virions enter the eclipse phase (5–12 hpi), before starting to replicate and infecting other cells; (**b**) IAV and immune response (IR) dynamics. The innate IR is mainly represented by interferon (IFN)-I and by natural killer (NK) cells, whereas the adaptive IR is mainly driven by cytotoxic CD8${}^{+}$ T cells (CTLs) and antibodies (Abs). Days post infection is abbreviated with dpi.

IAV infects mainly epithelial cells of the upper respiratory tract. However, IAV can infect cells of the lower respiratory tract in more severe cases [12,13,14]. Typical symptoms of IAV include fever, muscle aches, fatigue, runny nose and sore throat [15]. IAV infections are self-limited due to the development of a protective immune response (IR) [16]. An IAV infection starts when virions [17] enter the upper portion of the respiratory tract. Figure 1a shows major IAV infection events within a host. In the first phase, the binding and fusion capacity of the IAV hemagglutinin protein [18] facilitates virion adhesion to the receptors of the epithelial cell surface (*binding*). Approximately 20 min after infection, the virion is endocytosed inside the epithelial cell (*endocytosis*) [19]. Once inside the cell, the viral RNA is released and used as template to produce new virions, which are subsequently released into the extracellular environment (*budding*). The period between successful infection of the cell and the virus release is denominated the “*eclipse phase*” [20,21], which ranges between 5 and 12 hours post infection (hpi) [9,22]. After that, the new virions can start to infect other cells. The loss of virions can be attributed either to loss of virus infectivity or clearance by the immune system.

IAV replication peaks around 2–3 days post infection (dpi). Viral clearance is observed in most of the cases between 5 and 10 dpi [23]. In IAV infections, pathogen specific antibodies (Abs) and cytotoxic CD8${}^{+}$ T cells (CTLs) are detectable after 5 dpi and peak at 7 and 10 dpi respectively [24]. However, Immunoglobulin (Ig)G Abs peak later around 25 dpi [25]. Natural killer (NK) cells and interferon (IFN)-I are the main components of the innate immune system attributed to play a role in controlling IAV infections [26]. These components provide early immune containment of the IAV infection. Figure 1b depicts the dynamics of IAV infection and the principal elements of the immune system. Mathematical models capable of quantifying the main immune factors involved in the responses to IAV infections have the potential to provide critical information to advance our understanding of the immunity to this virus. Importantly, this information can aid in novel vaccine design and/or lead to strategies that limit complications such as bacterial coinfections. Additionally, the information derived from these models can suggest strategies to boost the IRs to IAV vaccines in populations that are prone to complications (e.g., the elderly).

## 2. Mathematical Models of IAV Infections

### 2.1. In Vivo Systems

The first mathematical model to describe IAV dynamics was developed in 1976 by Larson *et al.* [27]. The model was fitted to viral titer data of mice infected with IAV (H3N2). After thirty years without modeling efforts, a work that described the IAV infection dynamics was presented by Baccam *et al.* [26], which adopted the well-known target cell model [28,29,30].

**Figure 2:**
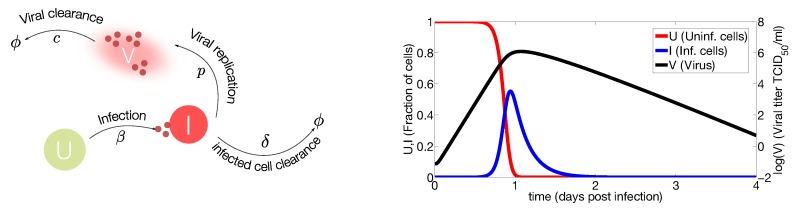
Target cell model. (**Left**) IAV (V) infects susceptible cells (U) with rate β. Infected cells are cleared with rate δ. Once cells are productively infected (I), they release virus at rate p and virus particles are cleared at rate c. The symbol ϕ represents clearance; (**Right**) Computational simulations of the target cell model. Parameter values used for model simulation are taken from [26]. The susceptible cells (red line) are rapidly infected while the virus (black line) and infected cells (blue line) peak at day one approximately. The viral growth is limited by the number of susceptible cells, decreasing the viral load and the number of infected cells to undetectable levels.

The target cell model is represented by susceptible cells (U), infected cells (I) and virus (V) as shown in Figure 2.

(1) $$\dot{\text{U}}=-\beta \text{UV}\;\;\;\;\;\;\;$$

(2) $$\dot{\text{I}}=\beta \text{UV}-\delta \text{I}$$

(3) $$\dot{\text{V}}=\text{pI}-\text{cV}\;\;\;\;\;$$

Several mathematical works have tried to model the eclipse phase *in vivo* [26] and *in vitro* [31,32]. These models have aimed at representing the time frame of the infection more adequately. This has resulted in an additional state, in which newly infected cells rest in a latent phase before becoming productively infected cells (I). Thus, the model in Equations (1)–(3) with the eclipse phase can be represented as follows:

(4) $$\begin{array}{cc} \dot{\text{U}} & =-\beta \text{UV} \end{array}$$

(5) $$\begin{array}{cc} \dot{{\text{E}}^{{}^{\prime }}} & =\beta \text{UV}-\text{k}{\text{E}}^{{}^{\prime }} \end{array}$$

(6) $$\begin{array}{cc} \dot{\text{I}} & =\text{k}{\text{E}}^{{}^{\prime }}-\delta \text{I} \end{array}$$

(7) $$\begin{array}{cc} \dot{\text{V}} & =\text{pI}-\text{cV} \end{array}$$

where ${\text{E}}^{{}^{\prime }}$ represents the cells in the eclipse phase, which can become productively infected at rate k. In Holder and Beauchemin [32], the authors considered different time distributions for modeling the eclipse phase and viral release by infected cells. The different mathematical model formulations were fitted with *in vitro* data. The results showed that the time distribution forms of the eclipse phase and viral release directly affect the parameter estimation. Baccam *et al.* [26] fitted the model Equation (4)–(7) with data from human volunteers infected with IAV A/HongKong/123/77 (H1N1). The estimated biological parameters e.g., viral clearance and cell half-life provided quantitative means of the viral infection dynamics. However, as argued by the authors, the parameter values should be used with caution due to identifiability problems (parameters can not be estimated in a unique way from the respective experimental data). In a similar direction Handel *et al.* [33] applied the target cell model to human influenza data to assess the emergence of resistance to neuraminidase inhibitors. However, this study also raised identifiability issues and confidence intervals were not provided. Recently, Dobrovolny *et al.* [34] adopted a double target cell model [35] with the eclipse phase to predict the efficacy of neuraminidase inhibitors on uncomplicated and more severe infections inside a host. For this purpose, the authors considered two different types of target cells (default and secondary). The first, is the fraction of cells available to IAV infection; meanwhile the second type of cell is the one accessible for severe IAV infections. The model was capable of mimicking the dynamics of uncomplicated IAV infection, including the IR in the secondary cell population dynamics. In another study, Petrie *et al.* [36] investigated parameter uncertainty using the target cell model and explored the possible reduction of parameter uncertainty fitting by measuring both infectious (via tissue infection culture dose 50 (TCID${}_{50}$)) and total viral load (via reverse transcription polimerase chain reaction (RT-PCR)). In addition, Petrie *et al.* [36] revealed that the variation in TCID${}_{50}$ assay sensitivity and calibration may affect the parameter estimation.

### 2.2. Mathematical Models Including the Immune Response

Although the target cell model can predict the dynamics of IAV infection without considering the host IR, control of the IR on the viral infection is fundamental to viral clearance [37]. To better understand the factors shaping the IAV infection course, a comprehensive model should incorporate the IR dynamics and its interactions with IAV. Numerous mathematical models have been introduced to investigate and predict the dynamics of the IR during IAV infections [11]. The first model was proposed by Bocharov and Romanyukha [38], which considered different mediators of the immune system. This study uncovered that CTLs and Abs are the main players controlling IAV infections. Hancioglu *et al.* [39] adopted the same modeling approach as Bocharov and Romanyukha [38], concluding that the initial viral load influenced disease severity. The target cell model was extended by Baccam *et al.* [26] to include the role of IFN-I. The IFN-I dynamics were modeled with the equation $\frac{\text{dF}}{\text{dt}}=\text{s}{\text{E}}^{{}^{\prime }}\left(\text{t}-\tau \right)-\alpha \text{F}$, where F is the level of the IFN-I, s is the secretion rate of IFN-I by infected cells, α is the IFN-I clearance rate and τ the lag period necessary to secret IFN-I. In this model, the influence of IFN-I is assumed to inhibit the viral replication rate through the fractional form $\frac{\text{p}}{1+\epsilon \text{F}}$, where p is the viral replication rate and ϵ is the effectiveness of IFN-I. Although, problems of parameter identifiability arose, the mathematical model including IFN-I dynamics was able to describe the double peaks present in some viral titer data. The role of IFN-I in preventing the infection of new cells was also investigated in [39,40,41,42,43]. In these works, the target cell model was extended taking into account another compartment where the cells remain refractory to IAV infection. In Hancioglu *et al.* [39], the dynamics of IFN-I were considered to promote the resistance of epithelial cells to IAV infection. An increased production rate of IFN-I and induction to resistance rate were fundamental to control disease duration and damage. Saenz *et al.* [41], modeled viral and IFN-I dynamics using data from equine IAV infection, revealing the extensive role of innate immunity in controlling the rapid peak of IAV infection. Using the same equine IAV infection data but with less parameters than [41], Pawelek *et al.* [40] included the IFN-I response in the target cell model. It was shown that the rapid and viral decline after the peak can be explained by the killing of infected cells mediated by IFN-I activated cells, such as NK cells. Hernandez-Vargas *et al.* [42] revealed that proinflammatory cytokines levels (IFN-α, IFN-γ, tumor necrosis factor (TNF)-α) could be responsible for slowing down viral growth in aged mice, limiting the activation of CTLs and causing the impaired IR to IAV infection reported in elderly. Recently, the mechanisms of innate IR in IAV reinfection data were investigated in [43,44]. Cao *et al.* [43] compared different in-host reinfection models including different IFN-I control mechanisms of viral infection on *in vivo* data. The authors revealed that a model that included a cell in an IFN-induced state-of-resistance cannot explain the observed viral hierarchy. Moreover, the authors postulated that the dynamics of secondary infection strongly depend upon the inter-exposure time.

Another relevant component of the innate IR is driven by NK cells [45,46]. Canini and Carrat [45] introduced a mathematical model with NK activation induced by IFN-I. Activated NK cells (N) follow the equation $\frac{\text{dN}}{\text{dt}}=\text{F}-\rho \text{N}$, where ρ is the NK cell death rate and F the level of IFN-I. Fitting of the viral kinetic and symptom data showed a correlation between within-host parameters and illness course. In addition, the adaptive IR dynamics directed by CTLs and Abs have been investigated in different works, all of which have concluded that CTLs play a relevant role in the clearance of the viral infection [11,25,34,39,47,48,49,50,51,52]. Lee *et al.* [52] developed a mathematical model quantifying the relations between viral replication and adaptive immunity. This mathematical model predicted that (i) CD4${}^{+}$ T cells have a prominent role in antibody persistence; (ii) antiviral drugs need to be administered within 2 dpi; and (iii) CTLs in the lung are as effective as neutralizing Abs for virus clearance, when present at the time of challenge. A relevant study dissecting the early and late IAV infection phases was conducted by Miao *et al.* [25]. In that study, the data of the IRs were fitted with a splines method [53], which was then used as inputs of the viral dynamics. The authors estimated important kinetic parameters (e.g., the half-life of infected cells, the infection rate of target cells) revealing the crucial role of CTLs in clearing the infection. In the same year, Handel *et al.* [47] identified two models with and without IR. Using a simplified Abs response, the authors argued that the data considered were not sufficient to discriminate the effect of the IRs. Tridane and Kuang [54] investigated viral clearance, modeling the interactions between IAV-infected epithelial cells and CTLs. The authors assumed two models for CTLs response (logistic and threshold growth). The systematic analysis showed that both model, fail to represent the mechanisms of viral clearance. An elegant work by Dobrovolny *et al.* [11] assessed the ability of mathematical models including different components of IR to predict the viral titer course. The authors compared these models with “knockout” experimental data, finding that no single model was able to explain the different viral kinetics. Recently a within-host mathematical model was developed by Price *et al.* [51], merging the innate and adaptive IR dynamics with inflammation. Authors showed a positive effect of controlled inflammation by IR.

### 2.3. In Vitro Systems

The complexity of the host immune system poses a significant challenge for the identification of critical factors involved in the interaction with IAV. This complexity has limited the exploration of some of their basic biological functions. An *in vitro* framework can simplify the system providing a small number of components to study. For example, key biological aspects can be inferred by analyzing viral infections of cell cultures (*in vitro*). In fact, a large array of mathematical models, focused on understanding the interactions between the virus and target cells, have been developed using *in vitro* generated data. For instance, Moehler *et al.* [55] applied the target cell model to include cell death as well as replenishment of target cells. This model described the viral kinetics of the equine IAV (A/Equi 2 (H3N8), Newmarket 1/93) in Madin-Darby canine kidney (MDCK) cells (microcarrier culture) and concluded that the number of available target cells is fundamental for viral growth.

Beauchemin *et al.* [31] explored different variants of the target cell model. In the experiments, the dynamics of MDCK cell infection by IAV (A/Albany/1/98 (H3N2)) were studied in the presence of amantadine and showed that the efficacy of this drug to block the infection of target cells was 56%–74%. In Schulze-Horsel *et al.* [56] infection of MDCK cells with different IAV strains was studied. The model described the dynamics of virus particle release (infectious virions and hemagglutinin content) and the parameters were estimated for different IAV strains. The results suggested that strains with slow initial infection dynamics but late induction of apoptosis resulted in higher viral yields. In Holder *et al.* [57], the authors compared fitness of the wild-type (WT) IAV (A/Brisbane/59/2007 (H1N1)) and a strain that acquired the oseltamivir-resistance mutation H275Y in its neuraminidase (NA) gene. Two different experimental settings were used: plaque assay and viral yields. The plaque assay indicated a higher fitness for the WT strain. In contrast, the viral yield assay suggested a higher fitness for the H275Y strain. Interestingly, *in silico* versions of these assays showed that the plaque assay was more sensitive for studying the duration of the eclipse phase, while the viral yield assay was sensitive for virus production. The different sensitivities of two assays could explain the conflicting results in terms of fitness. Later, Pinilla *et al.* [21] revealed that the principal effects of the H275Y substitution on the pandemic H1N1 (H1N1pdm09) strain were to lengthen in the mean eclipse phase of infected cells (from 6.6 to 9.1 h) and decrease (by 7-fold) the viral burst size. More recently, Paradis *et al.* [58] using the same model, compared the viral fitness of H275Y and I223V NA mutants with the pandemic H1N1 (H1N1pdm09) WT strain. The eclipse phase duration was longer for both mutants than the eclipse phase length of WT strain (by 2.5 and 3.6 h respectively). These works show the enormous benefits of mathematical modeling and how *in vitro* experimental data can support the development of a mathematical model and *vice versa*.

### 2.4. Data for Modeling: Scarce and Diverse

Table 1 presents the most recent list of works concerning mathematical models in IAV infections. Quantitative results can be largely affected by the host and the experimental setting. Animal models can allow more controlled experiments [18]. IAV infection kinetics have been studied in different hosts, including avian species (wild bird and domestic poultry), mammals (swine, pony, ferret, mouse, guinea pig, non-human primates) [18]. The mouse model is widely used mainly because of its low cost, availability of different strains and “knockouts” of genes that lead to deficiencies of specific immune populations or products (*i.e.*, T cells, cytokines, *etc.*) [59].

**Table 1:** Overview of the different mathematical models for IAV infection.

References	*In Vitro*	*In Vivo*		Host	Coinfection	Aging
		Innate	Adaptive			
Antia *et al.* [48]			√	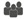		
Baccam *et al.* [26]		√		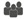		
Beauchemin *et al.* [31]	√			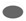		
Bocharov and Romanyukha [38]			√	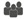		
Canini and Carrat [45]		√		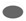		
Cao *et al.* [43]		√		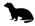		
Chen *et al.* [60]		√		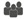		
Dobrovolny *et al.* [35]	√			Various		
Hancioglu *et al.* [39]		√		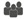		
Handel *et al.* [33]		√	√	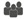		
Handel and Antia [49]			√	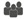		
[61]	√			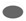		
Hernandez-Vargas *et al.* [42]		√	√	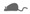		√
Holder *et al.* [57]	√			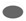		
Holder and Beauchemin [32]	√			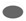		
Le *et al.* [50]			√	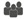		
Lee *et al.* [52]		√	√	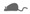		
Miao *et al.* [25]		√	√	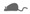		
Mitchell *et al.* [62]	√			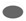		
Moehler *et al.* [55]	√			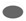		
Paradis *et al.* [58]	√			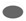		
Pawelek *et al.* [40]		√		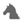		
Petrie *et al.* [36]		√		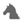		
Pinilla *et al.* [21]	√			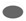		
Price *et al.* [51]		√	√	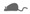		
Reperant *et al.* [63]		√	√	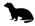		
Saenz *et al.* [41]		√		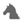		
Schulze-Horsel *et al.* [56]	√			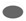		
Smith *et al.* [64]		√		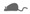	√	
Tridane and Kuang [54]			√	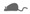		

However, it is worth mentioning that IAV transmission in mice is less efficient than in humans [18], and thus might not reflect well the infection in humans. Correspondingly, many mathematical models have derived the model parameters from murine experiments [10,25,27,42,47,52]. *In vitro* experimental data in cell lines (e.g., MDCK cells) also contributed valuable informations [55,57]. A major problem when comparing mathematical models is the difference between experimental settings in which the experiments are performed. For example, viral titers, as defined in many works, are provided in different units (e.g., RNA copies, plaque-forming units (PFU/mL), focus forming units (FFU/mL), TCID${}_{50}$ or egg infection dose 50 (EID${}_{50}$/mL)). Moreover, for the use of different units, knowing what they actually measure is critical for constructing reliable models and obtaining accurate parameter estimates (e.g., RNA copies include both infectious and non-infectious virions whereas TCID${}_{50}$ only indicates those infectious ones) [36]. However even in the case where experimental data present the same viral titer units, experiments conducted in different labs cannot be compared because the viral units are not standardized [58]. Moreover, Paradis *et al.* [58] suggested that even similar experiments performed in the same laboratory but at different time points cannot be compared. In addition to the noisy nature of biological measurement, the data used in mathematical models were not usually collected with a modeling purpose in mind. As a consequence, the data collected may not be optimal for identifying the model parameters. In particular, there are usually many measurements in the viral growth phase while measurements in the clearance phase are missing. These issues could be solved, if a close and iterative collaboration between biologists and modelers is established before the experimental design.

### 2.5. Parameter Estimation: A Continuous Challenge

Identification of infection kinetic parameters is one of the fundamental tasks to advance the knowledge of IAV infection mechanisms. These may help and allow experimentalists to test different scenarios. Nevertheless, estimating properly the parameters is a big challenge when the data are limited. In [9], the authors reviewed the parameters of IAV kinetics from several studies and showed a large variation of IAV infection kinetics parameters, which is the result of different experimental settings (e.g., *in vivo*, *in vitro*), different hosts and IAV strains.

**Figure 3:**
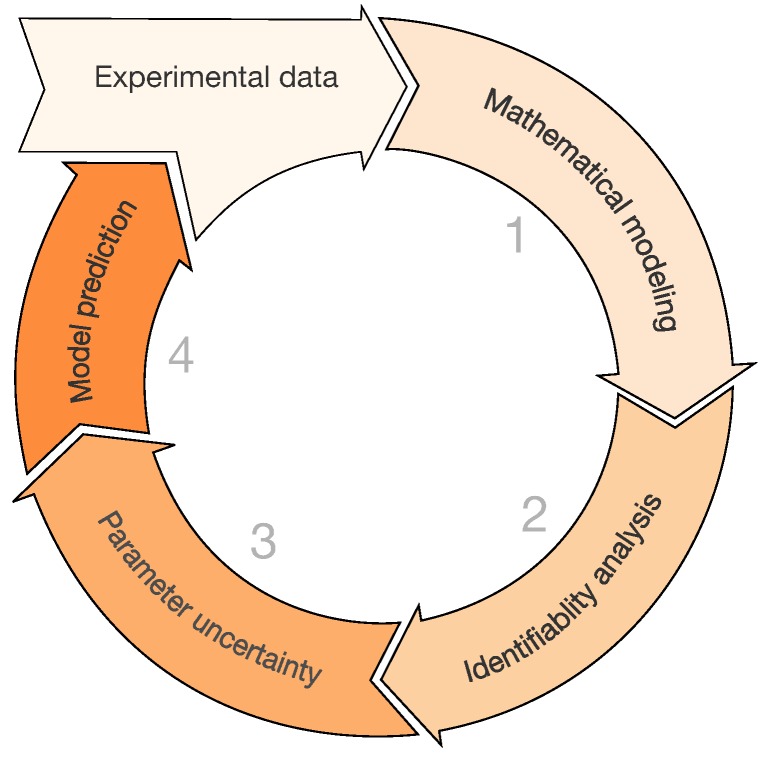
Mathematical modeling approach. From an experimental data set available, a mathematical model is developed/applied (**step 1**); Then, the identifiability analysis (**step 2**) should be carried out; Then, parameter uncertainty (**step 3**) is evaluated providing parameter confidence intervals. In this phase scatter plots can inform on the parameters relation and their influence on the mathematical model; Once reasonable parameter values are obtained, model prediction (**step 4**) can be performed generating new knowledge on the biological process and testing different scenarios.

Thus, parameter estimation procedures induce many questions on the robustness of the results. This highlights the problem in developing detailed mathematical models with a large number of parameters to be identified on a limited data set. Concurrently, another bottleneck is represented by the methodological tools used to perform parameter estimation. We overview briefly the mathematical modeling approach for model identification in Figure 3.

To generate a quantitative understanding and to explore particular hypotheses, the formulation of different mathematical models needs to be driven by the experimental data (**step 1**). Note that multiple models can provide the same fit with observed experimental data. Thus, it becomes necessary to choose between different models. The standard approach to model selection is first estimate all model parameters from the data, then select the model with the best-fit error and some penalty on model complexity (Akaike Information Criterion, Bayesian Information Criterion [65]).

After the model selection process, a key obstacle in order to obtain quantitative knowledge is the *identifiability* (**step 2**) of model parameters. A mathematical model is said to be identifiable when the parameter set can be uniquely determined. This can be achieved from the mathematical model structure (*structural*) and from the experimental data (*practical*). The *structural* and *practical* identifiability are necessary in mathematical models to reach significant predictions [66,67,68,69,70,71]. A very reliable method to test both the structural and practical identifiability is the profile likelihood method proposed by Raue *et al.* [72]. The idea behind this approach is to explore the parameter values, requiring for each parameter the optimization procedure of the cost function with respect to all other parameters. In particular, for each parameter, a range of values centered at the optimized value is generated in an adaptive manner. Re-optimization of the cost function with respect to the other parameters is performed for each value of the parameters. The aim of this approach is to detect directions where the likelihood flattens out [72].

Once the identifiability analysis is completed, *parameter uncertainty analysis* (**step 3**) is necessary to assess the large variability usually encountered in the biological data. The most frequent approach for parameter estimation is the bootstrap method. Bootstrapping is a statistic method for assigning measures of accuracy to estimates [73]. The nonparametric bootstrap considers data to be independent and identically distributed, whereas the parametric bootstrap requires imposing on the data a distribution assumption which is usually unknown. Bootstrap methods are frequently used as a conventional tool to take into account the uncertainty of the estimated parameter by calculating the confidence interval from bootstrap samples [10,25,42,57,74]. The bootstrap methods can be affected by the large variation of a few measurements or by the imposed distribution assumption that is usually unknown. As a result, the parameters confidence intervals can span a broad range [9]. Improvements of the bootstrap method in mathematical modeling have been proposed in [75,76,77]. Alternatively, the Bayesian approach could deal more efficiently with the parameters uncertainty, as well as the model prediction [78]. At the moment, there were only a few applications of the Bayesian methodology in mathematical modeling literature [79].

The outcome values of the bootstrap can serve to construct scatter plots. These are graphical sensitivity analysis methods, simple but useful tools to test the robustness of the results and highlight parameter dependence. Finally, once parameter distributions are inferred, one can test the predictive power of the mathematical model (**step 4**), generate new knowledge or hypotheses of the biological process of interest and guide the design of new experiments.

In summary, as presented in [80], future works on mathematical modeling of IAV infection should present the methodologies and parameter analysis to support the veracity of the estimations. In the next section, we exemplify how to conduct such mathematical modeling approach based on a mathematical model with the basic interactions between IR in IAV infections.

### 2.6. Case Study: Identification of a Mathematical Model of IAV Infection Including the Immune Response

**Figure 4:**
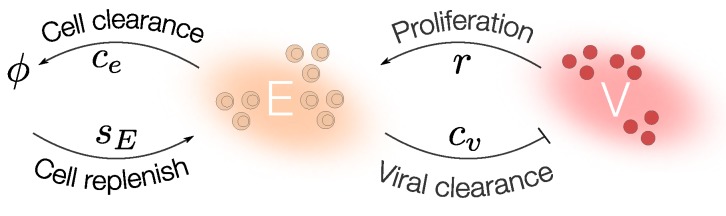
Viral infection model with CTLs response. IAV (V) induces CTLs (E) clonal expansion with a rate *r* which inhibits the viral replication through the clearance of the infected cell, this effect can be included in ${\text{c}}_{\text{v}}$. CTLs are replenished with rate ${\text{s}}_{\text{E}}$ and die with rate ${\text{c}}_{\text{e}}$.

Several studies investigated quantitatively the relevance of CTLs to clear IAV infections [25,39,40,41,42,52]. Due to the identifiability limitations to estimate the parameters in the target cell model only with viral load data, we propose a minimalistic model able to fit IAV and IR dynamics (Figure 4). In this example, we follow the mathematical modeling approach described in the previous section and schematized in Figure 3.

#### Step 1: Mathematical Modeling

We consider a mathematical model assuming that IAV (*V*) replication induces the proliferation of CTLs (*E*). We assume that the clearance of the infected cells by CTLs can be represented by the clearance of the virus. The dynamics of V and E can be described by the following equations:

(8) $$\begin{array}{cc} \dot{\text{V}} & =\text{pV}\left(1-\frac{\text{V}}{{\text{K}}_{\text{v}}}\right)-{\text{c}}_{\text{v}}\text{VE} \end{array}$$

(9) $$\begin{array}{cc} \dot{\text{E}} & =\text{rE}\left(\frac{\text{V}}{\text{V}+{\text{k}}_{\text{e}}}\right)-{\text{c}}_{\text{e}}\text{E}+{\text{s}}_{\text{E}} \end{array}$$

We define the CTLs replenishment rate ${\text{s}}_{\text{E}}={\text{c}}_{\text{e}}{\text{E}}_{0}$, where ${\text{E}}_{0}$ is the initial number of CTLs and ${\text{c}}_{\text{e}}$ is the half life of CTLs. The steady state should be satisfied to guarantee the CTLs homeostatic value $\text{E}={\text{s}}_{\text{E}}/{\text{c}}_{\text{e}}$ in the absence of viral infection (V=0). The CTLs proliferate at a rate r. We assume the activation of CTLs proliferation by V follows a Michaelis Menten growth with half saturation constant ${\text{k}}_{\text{e}}$. The V growth is modeled with a logistic function with maximum carrying capacity ${\text{K}}_{\text{v}}$ and growth rate p. V is cleared at a rate ${\text{c}}_{\text{v}}\text{E}$.

#### Step 2: Identifiability Analysis

**Figure 5:**
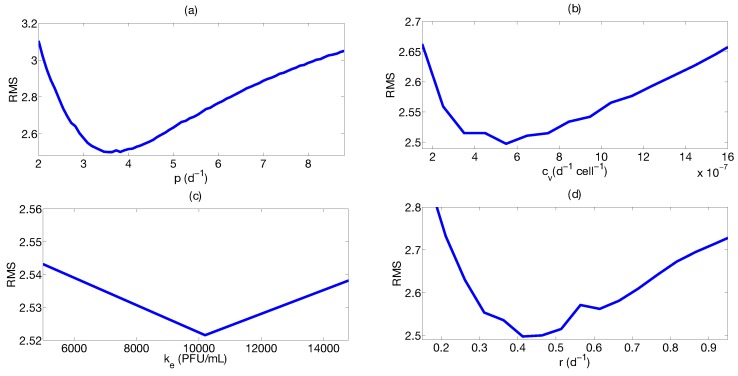
Profile likelihood for the model parameters. (**a**) p is the viral replication rate; (**b**) ${\text{c}}_{\text{v}}$ represents the viral clearance; (**c**) ${\text{k}}_{\text{e}}$ is the CTLs half saturation constant; (**d**) r represents the CTLs proliferation rate.

Here, we consider the murine data from [81] to estimate the model parameters. In this cross-sectional study, young and aged BALB/c mice were infected with 50 μL (50–100 PFU) of the IAV (H1N1) (PR8) . Lung viral titers were measured at different time points by plaque assays. Various components of the IR were also monitored. In this section, the viral and CTLs time course from the young mice are considered. To reduce the number of parameters to be identified, we fix the value of ${\text{c}}_{\text{e}}=2\times 10^{-2}$ (half life is approximately T=34 days) as reported by [82] and ${\text{K}}_{\text{v}}$ equal to the maximum value of the viral titer data in [81]. Therefore, the profile likelihood analysis can be computed on the unknown model parameters, this is shown in Figure 5. Note, that all the parameters present a profile likelihood with a minimum, implying that parameters are identifiable [72]. Therefore, the results suggest the possibility to infer model parameters from the available experimental data set.

#### Step 3: Parameter Uncertainty

After checking the identifiability properties of parameters, we proceed with the parameter uncertainty analysis. To this end, we consider the nonparametric bootstrap to obtain parameter estimates and 95% confidence intervals [76].

**Figure 6:**
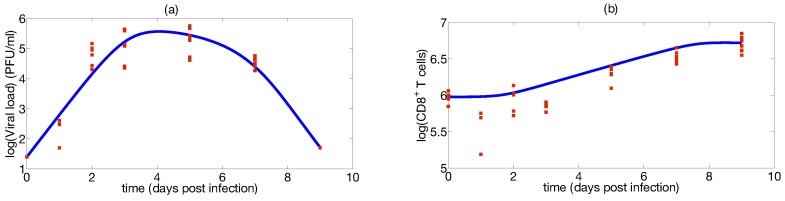
Viral infection model fitting: (**a**) The model fitting is shown with a blue line, viral load data is presented in red squares for mice infected with the IAV (H1N1) (PR8) strain; (**b**) the model fitting is shown in a blue line, the CTLs data is presented in red squares.

**Figure 7:**
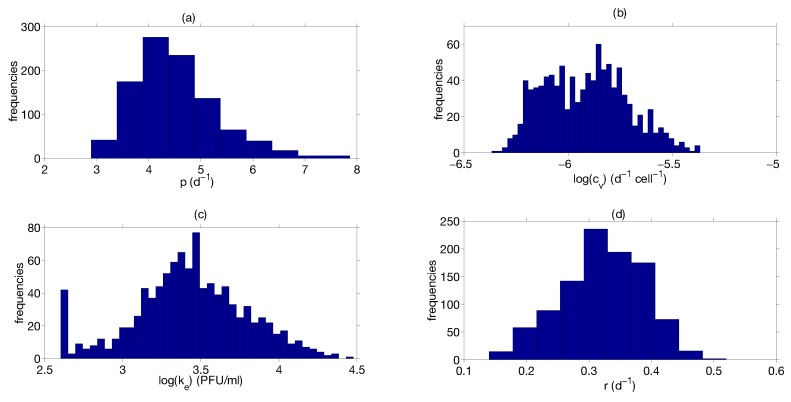
Nonparametric bootstrap results. The distributions of the nonparametric bootstrap obtained with 1000 samples for the model parameters (**a**) p; (**b**) ${\text{c}}_{\text{v}}$; (**c**) ${\text{k}}_{\text{e}}$; (**d**) r.

**Figure 8:**
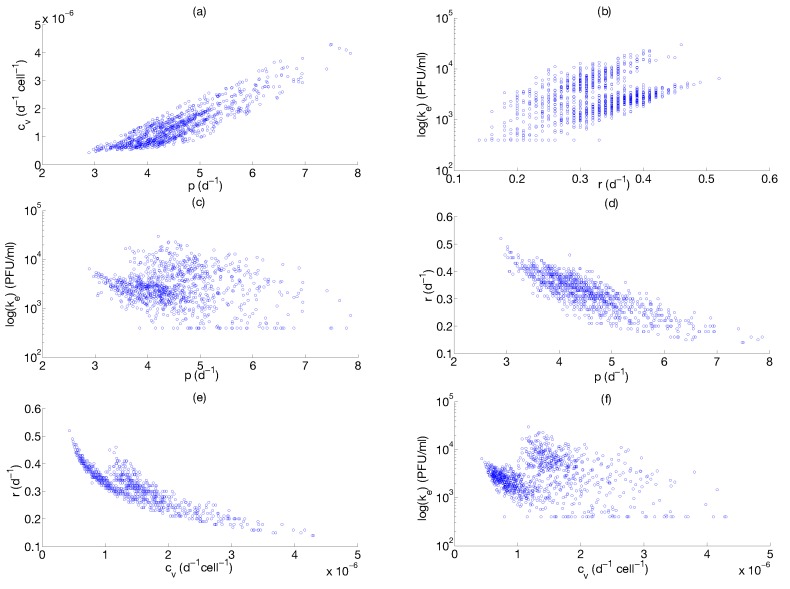
Scatter plot results. The scatter plots of (**a**) p-${\text{c}}_{\text{v}}$; (**b**) ${\text{k}}_{\text{e}}$-r; (**c**) ${\text{k}}_{\text{e}}$-p; (**d**) r-p; (**e**) r-${\text{c}}_{\text{v}}$, (**f**) ${\text{k}}_{\text{e}}$-${\text{c}}_{\text{v}}$. The numerical values are obtained from the nonparametric bootstrap distributions in Figure 7. The plots show dependencies between parameters p-${\text{c}}_{\text{v}}$, p-r, ${\text{c}}_{\text{v}}$-r.

A thousand different sampling repetitions from the data in [81] are performed. In each repetition, we draw a random sample from each time point and estimate the parameter. Model fitting and parameter distributions from nonparametric bootstrap are shown in Figure 6 and Figure 7, respectively. Parameter estimates and 95 % confidence intervals are shown in Table 2, respectively. The constraints for model parameter optimization, lower and upper bound, were broadly chosen to consider a large biological parameter space (see Table 2). The quality of fitting, as shown in Figure 6, indicates that the model is able to describe IAV infection and IR in murine data. The computed confidence intervals in Table 2 indicate a large interval only for the parameter ${\text{k}}_{\text{e}}$, while they result in a low extent for the remaining parameters. The large confidence interval of the parameter ${\text{k}}_{\text{e}}$ may be attributed to the flat profile likelihood observed in Figure 5c. Quantitative comparisons of parameters in Table 2 with the target cell model parameters are not possible due to the differences in model structures and units. Nevertheless, we can compare the viral production rate p in Table 2 with the viral growth rate presented in Smith *et al.* [83]. Authors proposed an approximation for the viral growth which is equivalent to the Figure 8 in the growth phase. Our p estimates [3.43; 6.08] ${\text{d}}^{-1}$ are in the same range of the estimate (6.59 ${\text{d}}^{-1}$) in [83]. Moreover the range of viral clearance rate ${\text{c}}_{\text{v}}\text{E}$ [1; 5.27] ${\text{d}}^{-1}$ is consistent to previous estimates [2.6; 15] ${\text{d}}^{-1}$ [26,40,42]. In order to explore possible parameter dependencies, we build scatter plots from the computed parameters values using the nonparametric bootstrap as it is shown in Figure 8.

**Table 2:** Model parameter estimates (median), 95% confidence intervals and constraints used in the optimization algorithm [84].

Parameter	Median	Confidence Interval (95%)	Constraints for Optimization Algorithm
$\text{p}[{\text{d}}^{-1}]$	4.4	[3.43 ; 6.08]	[1; 8]
${\text{c}}_{\text{v}}\left[{\text{d}}^{-1}\text{cel}{\text{l}}^{-1}\right]$	$1.24\times 10^{-6}$	[$6.1\times 10^{-7}$ ; $2.73\times 10^{-6}$]	[$5\times 10^{-8}$; $10^{-5}$ ]
$\text{r}[{\text{d}}^{-1}]$	0.33	[0.20 ; 0.42]	[0.01; 1]
${\text{k}}_{\text{e}}\left[\text{PFU}/\text{ml}\right]$	$2.7\times 10{}^{3}$	[$5.10\times 10{}^{2}$ ; $1.06\times 10^{4}$ ]	[$4\times 10^{2}$; $3\times 10^{4}$]

The scatter plots in Figure 8 show inter-dependencies between p-${\text{c}}_{\text{v}}$, p-r and ${\text{c}}_{\text{v}}$-r parameters. This implies that the estimation of one parameter influences the estimate of the others. For example, in the case of the parameters p-${\text{c}}_{\text{v}}$, increasing the value of the p increases the value of ${\text{c}}_{\text{v}}$, whereas increasing the value of p decreases the value of r. These parameter correlations could lead to possible parameter estimation problems. Moreover, we evaluate the dependence of model parameter estimates p, ${\text{c}}_{\text{v}}$, r, ${\text{k}}_{\text{e}}$ to viral carrying capacity ${\text{K}}_{\text{v}}$ (data not shown). The estimates of p and r showed no correlation with the parameter ${\text{K}}_{\text{v}}$, while there is a positive correlation of ${\text{c}}_{\text{v}}$ and ${\text{k}}_{\text{e}}$ estimates with ${\text{K}}_{\text{v}}$.

*Ad hoc* experiments to estimate parameter values could be a possible solution to alleviate parameter inter-dependencies and, therefore, estimate the remaining parameters more adequately. For example, in the previous work proposed by Pinilla *et al.* [21], virus titers were incubated without target cells and followed up to determine the remaining infectious titers. In this way, the approximate values of the viral clearance rate could be determined and provide a more accurate estimates for the whole set of kinetics parameters. The same procedure to reduce parameters inter-dependencies was followed in [31,58].

We want to close this section remarking how the mathematical modeling approach presented in Figure 3 can be useful to to advance our understanding of IAV and IR dynamics. We would like to point out that due to the nature of experimental data and the simple structure of our model further analysis are needed to provide more quantitative insights in the role of CTLs and IR to control IAV infection, as previous works pointed out [39,40,41,42,52].

## 3. Discussion and Future Perspectives

**Figure 9:**
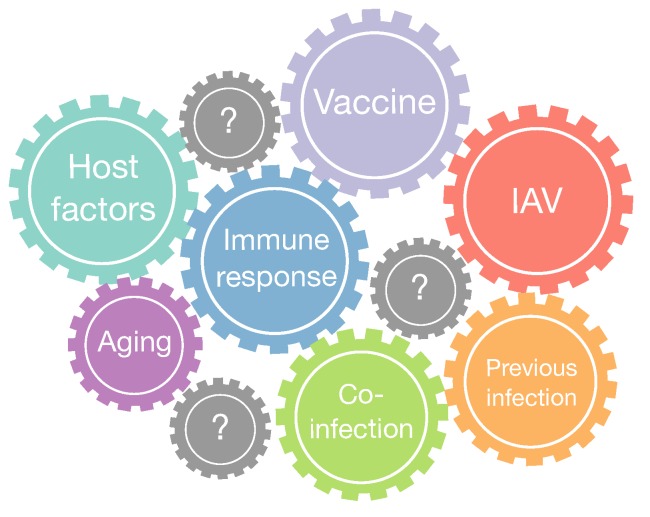
Main challenges in IAV infection. IAV infections facilitate secondary bacterial infections impairing the IR deputed to the bacterial clearance. The IAV infection is controlled by IR, which in turn is shaped by host genetic factors, previous infections, vaccination, and aging.

During last years, mathematical models of IAV have contributed to gain quantitative insights into viral infection mechanisms, within host dynamics and population scales [85]. These models considered different levels of complexity trying to explain the fundamental aspects of IAV infection and integrating most of the available biological knowledge. Next, we discuss what we consider are the main challenges for influenza research. These include: (i) bacterial coinfections; (ii) aging of the immune system and the role in IAV infection; (iii) challenges for influenza vaccination; (iv) host and IAV genetic factors (Figure 9). In particular, as illustrated in Table 1, for coinfection and aging only two mathematical modeling works have been carried out to date.

### 3.1. Bacterial Coinfection

Next to morbidity and mortality caused by IAV infection itself, enhanced susceptibility to bacterial coinfections represents a major risk. This was first recognized for the 1918/19 influenza outbreak (Spanish flu), as retrospective studies revealed that most of the several million of deaths were caused by severe complications due to secondary infections with bacterial pathogens [86]. A defined predisposition to life-threatening infections by bacteria (secondary infections) in IAV infected individuals was documented during epidemics in the 1950’s and 1960’s [87], as well as during the 2009 (H1N1) swine flu outbreak [88]. Altogether, epidemiological data clearly attest a high fatal synergism between IAV and many bacterial pathogens, such as *Streptococcus pneumoniae* (*S. pneumoniae*) [89].

For many decades, the straight-forward correlation between respiratory epithelial destruction caused by the viral infection and the facilitated adherence, growth and spread of bacterial pathogens remained the most accepted explanation for the increased susceptibility to secondary bacterial infections [90,91]. However, owing to a number of studies published over the last years, it is now largely understood that IAV infection also modulates the host’s immune system leaving it unable to mount an adequate anti-bacterial defense [92,93]. The proposed underlying mechanisms include the desensitization of alveolar macrophages [94], as well as the IFN-γ mediated suppression of their phagocytic function [95,96]. The recruitment and function of neutrophils, NK cells, and gamma-delta T cells have also been shown to be modulated during IAV infections [97,98,99]. Additionally, IFN-I has been identified as a major upstream mediator of some of these effects [99,100]. With regard to the respiratory epithelium, which hosts viral replication, IAV-mediated suppression of repair and regeneration processes [101], as well as the CD200-CD200R axis [102] have also been identified as players in the synergism between IAV and secondary bacterial pathogens.

Even though there have been rapid advances in our understanding of immune modulation through IAV, many questions still remain open. Enhanced susceptibility to bacterial infections can persist long after the virus has been cleared from the respiratory tract [94], but it remains largely elusive how and through which cell types these long term-effects are mediated. Furthermore, little attention has been paid to the question of whether certain viral strains are more efficient in predisposing the host to bacterial superinfection and, in turn, whether certain bacterial strains are favored, e.g., *S. pneumoniae* [92,103]. In this context, it is important to notice that primary IAV infections influence all aspects of bacterial pathogenesis, such as acquisition, transmission, colonization, replication, and adherence [103,104,105]. Even the live attenuated influenza vaccine (LAIV) increases the duration and density of *S. pneumoniae* nasopharyngeal carriage, which is a crucial prerequisite for invasive pneumococcal disease [106].

Despite the extensive research on the IAV role to facilitate bacterial colonization, the effects of *S. pneumoniae* on IAV infection dynamics are poorly understood. In McCullers [92], it is argued that the pneumococcal infection may strengthen IAV infection. The most plausible mechanisms proposed involve bacterial virulence factors interfering with the IR, such as, bacterial proteases facilitating endocytosis and synergy between bacterial and viral neuraminidase [92]. Ultimately, it will be essential to answer these open questions in order to complete our understanding of the synergism between IAV and secondary bacterial pathogens. This will in turn aid in the development of more effective prophylactic strategies and/or novel treatments.

Mathematical models can play an important role to unravel the key processes operating during coinfection. To the best of our knowledge, the only mathematical work to represent IAV-pneumococcus coinfection and the synergy between the virus and bacteria was developed by Smith *et al.* [64]. This work also addresses the role of the innate IR (alveolar macrophages) to clear bacteria. The down-regulation of bacterial clearance was modeled in [64] with a combination of two Michaelis-Menten functions including four parameters, assuming macrophages dynamics constant. We would like to highlight that even in the simpler form of bacterial clearance (${\text{c}}_{\text{B}}\text{B}$), the parameter ${\text{c}}_{\text{b}}$ is not identifiable. Then, taking into account a more complex form modeling the mechanisms of IR in the bacterial clearance as proposed in [64] will require the design of new experimental data. Thus, parameter values in [64] should be interpreted with caution. Further mathematical modeling research should include the interactions between macrophages, IFN-γ and CTLs, which can be important to uncover the underlying mechanisms between the innate and adaptive IR in bacterial clearance.

### 3.2. Aging of the Immune System and the Role in IAV Infections

The United Nations population division has predicted that by 2050 the number of elderly persons will increase from the current 600 million to nearly 2 billion worldwide. In developed countries, due to the rise in average life expectancy and reduced birth rates, 25% of the population is expected to be older than 65 years by 2050, making the elderly the fastest growing segment of the population [107]. Immunosenescence refers to the biological aging process associated with a progressive decline in systemic immunity and increased susceptibility to diseases such as cancer, autoimmune pathologies, chronic diseases (e.g., type 2 diabetes), poor responses to vaccination, and increased vulnerability to common infectious diseases [108]. The immune system becomes compromised, both quantitatively and qualitatively. A reduction in some cell populations and/or impaired responses has been described in murine models, and humans [81,109,110]. Both innate and adaptive IRs are affected. For instance, alterations in NK cells, NK T cells, dendritic cells (DC), monocytes and macrophages, and neutrophils have been described within the innate immune system [111,112,113]. On the adaptive side, immunosenescence has been associated with decreased clonal diversity of naive T cells, increased memory T cells, decreased CTLs function, decline of CD28 expression, increased number of regulatory T cells, and altered T cell signaling [81,114,115,116,117,118]. New insights into the changes induced by immunosenescence are coming from systems biology approaches [119,120]. Development of IRs and immunosenescence are complex biological processes and therefore not the result of isolated events, single proteins, chemicals, enzymes, or individual cell types. These changes are the coordinated effects of age-induced alterations in gene regulation and expression, signaling pathways within the cells, the interaction of various cell types, tissues, and biological networks. In sum, it is the synergistic effect of the individual parts that leads to impaired immunity and consequently immunosenescence caused by aging.

Mathematical models hold the potential to aid in elucidating correlates of protection for infectious diseases in the elderly. In Hernandez-Vargas *et al.* [42] age-related changes and their effects on influenza virus infection dynamics were examined. This work considered different IR components in young and aged mice: CTLs, NK cells, IFN-I, IFN-γ, and TNF-α. The fits of mathematical models to the experimental data from young and aged mice suggested that the increased levels of IFN-I, IFN-γ, and TNF-α (the “inflammaging” state) could promote the slower viral growth observed in aged mice, which consequently could limit the stimulation of immune cells and contributes to the reported impaired responses in the elderly. This study illustrated the advantages gained by the mathematical modeling approach to dissect quantitatively the role of the different IR mediators in young versus aged. However, more efforts are needed to uncover the age-related changes. It is important to consider that preexisting immunity to IAV, due to past infections/vaccination can directly alter the outcome described in [42].

### 3.3. Challenges for Influenza Vaccination

**Figure 10:**
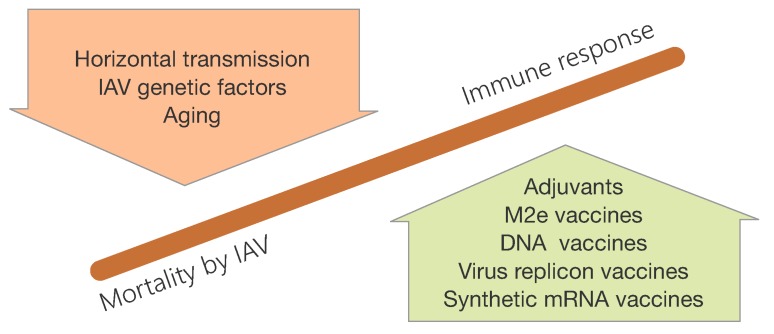
Emerging vaccination strategies. Novel vaccination strategies can for example (i) enhance mucosal IR against IAV reducing horizontal transmission and virus spread; (ii) display improved efficacy in poor responders such as elderly. Novel technologies will enable rapid production of emerging virus strains (e.g., synthetic mRNA or RNA replicon based vaccines) or universal vaccines covering major clades (e.g., designed hemagglutinins triggering broad neutralizing antibodies or vaccines triggering cross-protective CTL responses).

Although vaccination remains the cornerstone of influenza prophylaxis, the protection provided by seasonal vaccines is only partial, especially in high-risk groups such as the elderly [121,122]. Furthermore, new influenza virus strains arising from this zoonotic pathogen increase concerns about the threat of a new pandemia [123]. In this regard, the global spread of the (2009) (H1N1) pandemic virus within 2 months clearly demonstrated that current vaccine platforms cannot meet an emerging pandemia [124,125]. The global vaccine manufacturing capacity was inadequate and unable to supply enough vaccine doses promptly [126,127]. Thus, in order to increase vaccination efficacy huge efforts need to be taken to optimize vaccines (e.g., by co-admixing with adjuvants) which are already in the market and/or to develop new vaccination strategies [128] (Figure 10).

A possible strategy could be the development of a mucosal vaccine. Today, most of the licensed seasonal vaccines are administered via parenteral routes failing to promote mucosal immunity [129]. This is an important issue considering that the respiratory tract constitutes the port of entry for IAVs. In this context, mucosal immunization offers the potential to stimulate protective IRs at both the systemic level and the site of viral entry, e.g., by the induction of secretory IgA and CTLs activity [130,131,132]. However, current split and subunit vaccines do not elicit adequate IRs when administered via mucosal routes as they show reduced immunogenicity. On the other hand, the cold-adapted attenuated intranasal vaccine is only licensed for a very narrow subpopulation group. To overcome this constrain, adjuvants can be included in such mucosal vaccination strategies. Indeed, no mucosal adjuvant has been licensed worldwide so far. Mostly, this is related to either a lack of activity or safety concerns. For example, the widely used adjuvant aluminum hydroxide (alum) does not provide appropriate mucosal adjuvant activity, whereas the use of derivatives of the heat-labile enterotoxin of *Escherichia coli* administered by an intranasal route has been linked to Bell’s palsy [133,134].

Other novel adjuvants such as Polyethyleneimine (PEI) [135] or the novel nanoemulsion W(80)5EC were already tested in volunteers and elicited both systemic and mucosal immunity following a single intranasal vaccination [136]. It was shown that these compounds may significantly contribute towards the development of innovative mucosal vaccines against influenza, especially for high-risk groups. Beside the need of adjuvants, novel vaccination strategies would also be needed to stimulate efficient cellular IRs. For example, a vaccine based on the ectodomain of influenza matrix protein 2 (*M2e*) has been applied with adjuvants and carriers, or fused with flagellin [137]. The results of preclinical studies in animal models have shown that *M2e*-specific Abs limit IAV replication and reduce mortality rate [138]. Other multimeric vaccines based on proteins were tested in clinical trials benefited from Montanide adjuvant formulation [139,140]. The drawback remains in the limited amount of antigen available with protein vaccines, and their lower capacity for inducing cytotoxic defences compared with replicating viruses.

Efficacious immune defence against influenza virus requires both humoral and cytotoxic arms, as witnessed by the limited protection offered by current seasonal vaccines which induces mainly humoral responses [141]. Nevertheless, replicating vaccines were shown to be able to enhance both humoral and cytotoxic IRs. Thus, DNA vaccines as well as adenovirus and vaccinia virus vector-based vaccines have been developed. These vaccines are able to stimulate cross-clade neutralising antibodies and cell-mediated immunity (CMI) [142,143,144]. Another promising approach consists of self-replicating replicon-RNA(RepRNA) which is readily generated by synthetic means, and incorporated into biodegradable delivery vehicles. RepRNA replication increases the number of RNA templates and expressed antigens, inducing long-lasting humoral and CMI defences [145,146]. However, several new approaches are under development aiming in the generation of universal vaccines [147].

To the best of authors knowledge, modeling IAV vaccination as well as in-host vaccine effect has not been explored. Until now, mathematical models have focused mainly on the effect of the vaccines on population-level spread of IAV [148,149]. However, quantitative efforts are necessary to gain comprehension into the immunological signatures of vaccination, evaluating the efficacy of different vaccines, adjuvants, delivery systems and immunization routes [150,151,152]. This will only be possible with the help of quantitative models of affinity maturation of B cells in germinal centers [153] giving rise to Abs with high affinity to the respective pathogen [154]. However, affinity is not the only important Ab property in this context. The quantity and the affinity of the generated Abs are normally contradicting properties, and it might be interesting to test strategies to overcome this limitation of the overall strength of an Ab response [155] in the context of vaccinations. Furthermore, Ab cross-reactivity might be essential to determine the efficiency of a vaccine. Specificity and cross-reactivity of Abs emerging in response to monovalent and polyvalent vaccinations were investigated for the example of malaria [156]. A similar comparison of different influenza vaccines would enable us to address the age-dependent response to influenza vaccination in terms of both safety and efficacy [147], which represents a main unsolved challenge in vaccinology.

### 3.4. Host and IAV Genetic Factors

In the past, molecular mechanisms focusing on the IAV pathogen have been broadly investigated in influenza research. The processes of adhesion, entry, replication and assembly with respect to the individual mutations of the IAV were studied intensively. However, not only the viral but also host factors are crucial for the outcome of an influenza infection. Recently, crucial host factors could be identified (see Table 3).

Together with the help of animal models the complexity can be reduced, and the factor of interest can be studied under controlled circumstances. While transmission of IAV is mainly studied in ferrets, mice are advantageous since their immune system and genome are well characterized and research tools are available. Importantly, several aspects of the murine immune system are similar to the human one. Mouse models demonstrated a clear genetic effect on susceptibility to influenza A infection [157]. In addition to the availability of inbred mouse strains, targeted modifications of the genome resulting in transgenic mice are easily possible. A novel mouse population, the Collaborative Cross, with a genotypic variation comparable to the human population generated from eight founder strains might provide an excellent model system in future [158]. Preliminary studies using this population for IAV verified the significance of this approach [159,160]. Using such a systems approach this study contributes to a better understanding of underlying biological networks under host genetic control during an IAV infection [159].

**Table 3:** Host Genetic factors. Host factors identified having a crucial role to determine severity of IAV infection in different hosts.

Host Factors	Role
IFITM3	Restrict morbidity and mortality of IAV infection [161,162,163]
CPT2	Related complication as influenza-associated encephalopathy [164]
TMPRSS2	Resistance to IAV infection [165,166,167]

Due to the advent of modern “omics” techniques, a huge amount of data is becoming available in individuals. The gene network complexity can be only achieved with the help of systems-oriented approaches [168]. The deterministic framework with differential equations is the most widely used mathematical approach to describe key biological mechanisms. This is feasible only with high numbers of particles or species where the random fluctuations around the species concentrations are negligible [169]. Nevertheless, stochastic fluctuations are prominent in the presence of low mRNA copy numbers, becoming the “fingerprint” of gene regulatory network processes such as transcription and translation [170,171]. Thus, a stochastic modeling framework can play an important role to elucidate and dissect the host genetic mechanisms responsible for the IAV infections outcome (e.g., identify new drug targets, test the efficacy of treatments in a genetically heterogeneous model, identify susceptibility and resistance [169,170,172]).

## References

[1] World Health Organization (WHO) (2009). Influenza (Seasonal) Factsheet N 211.

[2] Lang P.O., Mendes A., Socquet J., Assir N., Govind S., Aspinall R. (2012). Effectiveness of influenza vaccine in aging and older adults: Comprehensive analysis of the evidence. Clin. Interv. Aging.

[3] Potter C.W. (2001). A history of influenza. J. Appl. Microbiol..

[4] Kilbourne E.D. (2006). Influenza pandemics of the 20th century. Emerg. Infect. Dis..

[5] World Health Organization (WHO) (2014). Influenza (Seasonal): Fact Sheet N^∘^211.

[6] Carrat F., Flahault A. (2007). Influenza vaccine: The challenge of antigenic drift. Vaccine.

[7] Hensley S.E. (2014). Challenges of selecting seasonal influenza vaccine strains for humans with diverse pre-exposure histories. Curr. Opin. Virol..

[8] Madhi S.A., Cutland C.L., Kuwanda L., Weinberg A., Hugo A., Jones S., Adrian P.V., van Niekerk N., Treurnicht F., Ortiz J.R. (2015). Influenza Vaccination of Pregnant Women and Protection of Their Infants. Obstet. Gynecol. Survey.

[9] Beauchemin C.A.A., Handel A. (2011). A review of mathematical models of influenza A infections within a host or cell culture: Lessons learned and challenges ahead. BMC Public Health.

[10] Smith A.M., Perelson A.S. (2011). Influenza A virus infection kinetics: Quantitative data and models. Syst. Biol. Med..

[11] Dobrovolny H.M., Reddy M.B., Kamal M.A., Rayner C.R., Beauchemin C.A. (2013). Assessing Mathematical Models of Influenza Infections Using Features of the Immune Response. PLoS ONE.

[12] De Wit E., Rasmussen A.L., Feldmann F., Bushmaker T., Martellaro C., Haddock E., Okumura A., Proll S.C., Chang J., Gardner D. (2014). Influenza virus A/Anhui/1/2013 (H7N9) replicates efficiently in the upper and lower respiratory tracts of cynomolgus macaques. mBio.

[13] Welliver T.P., Garofalo R.P., Hosakote Y., Hintz K.H., Avendano L., Sanchez K., Velozo L., Jafri H., Chavez-Bueno S., Ogra P.L. (2007). Severe Human Lower Respiratory Tract Illness Caused by Respiratory Syncytial Virus and Influenza Virus Is Characterized by the Absence of Pulmonary Cytotoxic Lymphocyte Responses. J. Infect. Dis..

[14] Van Riel D., Munster V.J., de Wit E., Rimmelzwaan G.F., Fouchier R.A.M., Osterhaus A.D.M.E., Kuiken T. (2006). H5N1 Virus Attachment to Lower Respiratory Tract. Science.

[15] Reeth K.V. (2000). Cytokines in the pathogenesis of influenza. Vet. Microbiol..

[16] Valkenburg S.A., Rutigliano J.A., Ellebedy A.H., Doherty P.C., Thomas P.G., Kedzierska K. (2011). Immunity to seasonal and pandemic influenza A viruses. Microbes Infect..

[17] Lindsley W.G., Noti J.D., Blachere F.M., Thewlis R.E., Martin S.B., Othumpangat S., Noorbakhsh B., Goldsmith W.T., Vishnu A., Palmer J.E. (2015). Viable Influenza A Virus in Airborne Particles from Human Coughs. J. Occup. Environ. Hyg..

[18] Oldstone M.B.A., Compans R.W. (2015). Influenza Pathogenesis and Control.

[19] Oguin T.H., Sharma S., Stuart A.D., Duan S., Scott S.A., Jones C.K., Daniels J.S., Lindsley C.W., Thomas P.G., Brown H.A. (2014). Phospholipase D facilitates efficient entry of influenza virus, allowing escape from innate immune inhibition. J. Biol. Chem..

[20] White D.O., Cheyne I.M. (1966). Early events in the eclipse phase of influenza and parainfluenza virus infection. Virology.

[21] Pinilla L.T., Holder B.P., Abed Y., Boivin G., Beauchemin C.A.A. (2012). The H275Y neuraminidase mutation of the pandemic A/H1N1 influenza virus lengthens the eclipse phase and reduces viral output of infected cells, potentially compromising fitness in ferrets. J. Virol..

[22] Horsfall F.L. (1954). On the reproduction of influenza virus quantitative studies with procedures which enumerate infective and hemagglutinating virus particles. J. Exp. Med..

[23] Banatvala J.E., Griffiths P., Schoub B., Mortimer P. (2009). Principles and Practice of Clinical Virology.

[24] Tamura S.i., Kurata T. (2004). Defense mechanisms against influenza virus infection in the respiratory tract mucosa. Jpn. J. Infect. Dis..

[25] Miao H., Hollenbaugh J.A., Zand M.S., Holden-Wiltse J., Mosmann T.R., Perelson A.S., Wu H., Topham D.J. (2010). Quantifying the early immune response and adaptive immune response kinetics in mice infected with influenza A virus. J. Virol..

[26] Baccam P., Beauchemin C.A.A., Macken C.A.A., Hayden F.G., Perelson A.S. (2006). Kinetics of influenza A virus infection in humans. J. Virol..

[27] Larson E.W., Dominik J.W., Rowberg A.H., Higbee G.A. (1976). Influenza virus population dynamics in the respiratory tract of experimentally infected mice. Infect. Immun..

[28] Nowak M.A., May R. (2000). Virus Dynamics: Mathematical Principles of Immunology and Virology.

[29] Perelson A.S. (2002). Modelling viral and immune system dynamics. Nat. Rev. Immunol..

[30] Ribeiro R.M., Lo A., Perelson A.S. (2002). Dynamics of hepatitis B virus infection. Microbes Infect..

[31] Beauchemin C.A.A., McSharry J.J., Drusano G.L., Nguyen J.T., Went G.T., Ribeiro R.M., Perelson A.S. (2008). Modeling amantadine treatment of influenza A virus *in vitro*. J. Theor. Biol..

[32] Holder B.P., Beauchemin C.A.A. (2011). Exploring the effect of biological delays in kinetic models of influenza within a host or cell culture. BMC Public Health.

[33] Handel A., Longini I.M., Antia R. (2007). Neuraminidase inhibitor resistance in influenza: Assessing the danger of its generation and spread. PLoS Comput. Biol..

[34] Dobrovolny H.M., Gieschke R., Davies B.E., Jumbe N.L., Beauchemin C.A.A. (2011). Neuraminidase inhibitors for treatment of human and avian strain influenza: A comparative modeling study. J. Theor. Biol..

[35] Dobrovolny H.M., Baron M.J., Gieschke R., Davies B.E., Jumbe N.L., Beauchemin C.A.A. (2010). Exploring cell tropism as a possible contributor to influenza infection severity. PLoS ONE.

[36] Petrie S.M., Guarnaccia T., Laurie K.L., Hurt A.C., McVernon J., McCaw J.M. (2013). Reducing Uncertainty in Within-Host Parameter Estimates of Influenza Infection by Measuring Both Infectious and Total Viral Load. PLoS ONE.

[37] Doherty P.C., Turner S.J., Webby R.G., Thomas P.G. (2006). Influenza and the challenge for immunology. Nat. Immunol..

[38] Bocharov G., Romanyukha A. (1994). Mathematical model of antiviral immune response III. Influenza A virus infection. J. Theor. Biol..

[39] Hancioglu B., Swigon D., Clermont G. (2007). A dynamical model of human immune response to influenza A virus infection. J. Theor. Biol..

[40] Pawelek K.A., Huynh G.T., Quinlivan M., Cullinane A., Rong L., Perelson A.S. (2012). Modeling within-host dynamics of influenza virus infection including immune responses. PLoS Comput. Biol..

[41] Saenz R.A., Quinlivan M., Elton D., Macrae S., Blunden A.S., Mumford J.A., Daly J.M., Digard P., Cullinane A., Grenfell B.T. (2010). Dynamics of influenza virus infection and pathology. J. Virol..

[42] Hernandez-Vargas E.A., Wilk E., Canini L., Toapanta F.R., Binder S.C., Uvarovskii A., Ross T.M., Guzmán C.A., Perelson A.S., Meyer-Hermann M. (2014). Effects of aging on influenza virus infection dynamics. J. Virol..

[43] Cao P., Yan A.W., Heffernan J.M., Petrie S., Moss R.G., Carolan L.A., Guarnaccia T.A., Kelso A., Barr I.G., McVernon J. (2015). Innate Immunity and the Inter-exposure Interval Determine the Dynamics of Secondary Influenza Virus Infection and Explain Observed Viral Hierarchies. PLoS Comput. Biol..

[44] Laurie K.L., Guarnaccia T.A., Carolan L.A., Yan A.W., Aban M., Petrie S., Cao P., Heffernan J.M., McVernon J., Mosse J. (2015). The time-interval between infections and viral hierarchies are determinants of viral interference following influenza virus infection in a ferret model. J. Infect. Dis..

[45] Canini L., Carrat F. (2011). Population modeling of influenza A/H1N1 virus kinetics and symptom dynamics. J. Virol..

[46] Hernandez-Vargas E.A., Meyer-Hermann M. Innate immune system dynamics to influenza virus. Proceedings of the 8th IFAC Symposium on Biological and Medical Systems.

[47] Handel A., Longini I.M., Antia R. (2010). Towards a quantitative understanding of the within-host dynamics of influenza A infections. J. Royal Soc. Interface Royal Soc..

[48] Antia R., Bergstrom C.T., Pilyugin S.S., Kaech S.M., Ahmed R. (2003). Models of CD8+ responses: 1. What is the antigen-independent proliferation program. J. Theor. Biol..

[49] Handel A., Antia R. (2008). A simple mathematical model helps to explain the immunodominance of CD8 T cells in influenza A virus infections. J. Virol..

[50] Le D., Miller J.D., Ganusov V.V. (2014). Mathematical modeling provides kinetic details of the human immune response to vaccination. Front. Cell. Infect. Microbiol..

[51] Price I., Mochan-Keef E.D., Swigon D., Ermentrout G.B., Lukens S., Toapanta F.R., Ross T.M., Clermont G. (2015). The inflammatory response to influenza A virus (H1N1): An experimental and mathematical study. J. Theor. Biol..

[52] Lee H.Y., Topham D.J., Park S.Y., Hollenbaugh J., Treanor J., Mosmann T.R., Jin X., Ward B.M., Miao H., Holden-Wiltse J. (2009). Simulation and prediction of the adaptive immune response to influenza A virus infection. J. Virol..

[53] De Boor C. (2001). A Practical Guide to Splines.

[54] Tridane A., Kuang Y. (2010). Modeling the interaction of cytotoxic T lymphocytes and influenza virus infected epithelial cells. Math. Biosci. Eng..

[55] Moehler L., Flockerzi D., Sann H., Reichl U. (2005). Mathematical model of influenza A virus production in large-scale microcarrier culture. Biotechnol. Bioeng..

[56] Schulze-Horsel J., Schulze M., Agalaridis G., Genzel Y., Reichl U. (2009). Infection dynamics and virus-induced apoptosis in cell culture-based influenza vaccine production Flow cytometry and mathematical modeling. Vaccine.

[57] Holder B.P., Simon P., Liao L.E., Abed Y., Bouhy X., Beauchemin C.A.A., Boivin G. (2011). Assessing the *in vitro* fitness of an oseltamivir-resistant seasonal A/H1N1 influenza strain using a mathematical model. PLoS ONE.

[58] Paradis E.G., Pinilla L.T., Holder B.P., Abed Y., Boivin G., Beauchemin C.A.A. (2015). Impact of the H275Y and I223V Mutations in the Neuraminidase of the 2009 Pandemic Influenza Virus *in Vitro* and Evaluating Experimental Reproducibility. PLoS ONE.

[59] Reperant L.A., Kuiken T., Osterhaus A.D. (2012). Adaptive pathways of zoonotic influenza viruses: From exposure to establishment in humans. Vaccine.

[60] Chen S., You S., Liu C., Chio C., Liao C. (2012). Using experimental human influenza infections to validate a viral dynamic model and the implications for prediction. Epidemiol. Infect..

[61] Heldt F.S., Frensing T., Pflugmacher A., Gröpler R., Peschel B., Reichl U. (2013). Multiscale modeling of influenza A virus infection supports the development of direct-acting antivirals. PLoS Comput. Biol..

[62] Mitchell H., Levin D., Forrest S., Beauchemin C.A.A., Tipper J., Knight J., Donart N., Layton R.C., Pyles J., Gao P. (2011). Higher level of replication efficiency of 2009 (H1N1) pandemic influenza virus than those of seasonal and avian strains: Kinetics from epithelial cell culture and computational modeling. J. Virol..

[63] Reperant L.A., Kuiken T., Grenfell B.T., Osterhaus A.D. (2014). The immune response and within-host emergence of pandemic influenza virus. Lancet.

[64] Smith A.M., Adler F.R., Ribeiro R.M., Gutenkunst R.N., McAuley J.L., McCullers J.A., Perelson A.S. (2013). Kinetics of coinfection with influenza A virus and Streptococcus pneumoniae. PLoS Pathog..

[65] Burnham K.P., Anderson D.R. (2004). Multimodel inference understanding AIC and BIC in model selection. Sociol. Methods Res..

[66] Li P., Vu Q.D. (2013). Identification of parameter correlations for parameter estimation in dynamic biological models. BMC Syst. Biol..

[67] Xia X., Moog C. (2003). Identifiability of nonlinear systems with application to HIV/AIDS models. IEEE Trans. Autom. Control.

[68] Miao H., Xia X., Perelson A.S., Wu H. (2011). On Identifiability of Nonlinear Ode Models and Applications in Viral Dynamics. SIAM Rev. Soc. Ind. Appl. Math..

[69] Chis O.T., Banga J.R., Balsa-Canto E. (2011). Structural identifiability of systems biology models: A critical comparison of methods. PLoS ONE.

[70] Brun R., Reichert P., Kuensch H.R. (2001). Practical identifiability analysis of large environmental simulation models. Water Resour. Res..

[71] Prill R.J., Marbach D., Saez-Rodriguez J., Sorger P.K., Alexopoulos L.G., Xue X., Clarke N.D., Altan-Bonnet G., Stolovitzky G. (2010). Towards a rigorous assessment of systems biology models: The DREAM3 challenges. PLoS ONE.

[72] Raue A., Kreutz C., Maiwald T., Bachmann J., Schilling M., Klingmüller U., Timmer J. (2009). Structural and practical identifiability analysis of partially observed dynamical models by exploiting the profile likelihood. Bioinform. (Oxf. Engl.).

[73] Efron B., Tibshirani R.J. (1994). An Introduction to the Bootstrap.

[74] Nguyen V.K., Binder S.C., Boianelli A., Meyer-Hermann M., Hernandez-Vargas E.A. (2015). Ebola Virus Infection Modelling and Identifiability Problems. Front. Microbiol..

[75] Ma S., Kosorok M.R. (2005). Robust semiparametric M-estimation and the weighted bootstrap. J. Multivar. Anal..

[76] Xue H., Miao H., Wu H. (2010). Sieve estimation of constant and time-varying coefficients in nonlinear ordinary differential equation models by considering both numerical error and measurement error. Ann. Stat..

[77] Vinod H.D. (2013). Maximum Entropy Bootstrap Algorithm Enhancements. Available at SSRN 2285041.

[78] Lesaffre E., Lawson A.B. (2012). Bayesian Biostatistics.

[79] Raue A., Kreutz C., Joachim Theis F., Timmer J. (2013). Joining forces of Bayesian and frequentist methodology: A study for inference in the presence of non-identifiability. Philos. Trans. Ser. A.

[80] Chou I.C., Voit E.O. (2009). Recent developments in parameter estimation and structure identification of biochemical and genomic systems. Math. Biosci..

[81] Toapanta F.R., Ross T.M. (2009). Impaired immune responses in the lungs of aged mice following influenza infection. Respir. Res..

[82] McDonagh M., Bell E. (1995). The survival and turnover of mature and immature CD8 T cells. Immunology.

[83] Smith A.M., Adler F.R., McAuley J.L., Gutenkunst R.N., Ribeiro R.M., McCullers J.A., Perelson A.S. (2011). Effect of 1918 PB1-F2 expression on influenza A virus infection kinetics. PLoS Comput. Biol..

[84] Storn R., Price K. (1997). Differential evolution—A simple and efficient heuristic for global optimization over continuous spaces. J. Global Optim..

[85] Murillo L.N., Murillo M.S., Perelson A.S. (2013). Towards multiscale modeling of influenza infection. J. Theor. Biol..

[86] Morens D.M., Taubenberger J.K., Fauci A.S. (2008). Predominant role of bacterial pneumonia as a cause of death in pandemic influenza: Implications for pandemic influenza preparedness. J. Infect. Dis..

[87] McCullers J.A. (2006). Insights into the interaction between influenza virus and pneumococcus. Clin. Microbiol. Rev..

[88] Louie J., Jean C., Chen T., Park S., Ueki R., Harper T., Chmara E., Myers J., Stoppacher R., Catanese C. (2009). Bacterial coinfections in lung tissue specimens from fatal cases of 2009 pandemic influenza A (H1N1)-United States, May-August 2009. Morb. Mortal. Wkly. Rep..

[89] Chertow D.S., Memoli M.J. (2013). Bacterial coinfection in influenza: A grand rounds review. JAMA.

[90] Louria D.B., Blumenfeld H.L., Ellis J.T., Kilbourne E.D., Rogers D.E. (1959). Studies on influenza in the pandemic of 1957-1958. II. Pulmonary complications of influenza. J. Clin. Investig..

[91] Martin C.M., Kunin C.M., Gottlieb L.S., Barnes M.W., Liu C., Finland M. (1959). Asian Influenza A in Boston. 1957–1958: I. Observations in Thirty-Two Influenza-Associated Fatal Cases. AMA Arch. Intern. Med..

[92] McCullers J.A. (2014). The co-pathogenesis of influenza viruses with bacteria in the lung. Nat. Rev. Microbiol..

[93] Robinson K.M., Kolls J.K., Alcorn J.F. (2015). The immunology of influenza virus-associated bacterial pneumonia. Curr. Opin. Immunol..

[94] Didierlaurent A., Goulding J., Patel S., Snelgrove R., Low L., Bebien M., Lawrence T., van Rijt L.S., Lambrecht B.N., Sirard J.C. (2008). Sustained desensitization to bacterial Toll-like receptor ligands after resolutionof respiratory influenza infection. J. Exp. Med..

[95] Sun K., Metzger D.W. (2008). Inhibition of pulmonary antibacterial defense by interferon-γ during recovery from influenza infection. Nat. Med..

[96] Stegemann-Koniszewski S., Gereke M., Orrskog S., Lienenklaus S., Pasche B., Bader S.R., Gruber A.D., Akira S., Weiss S., Henriques-Normark B. (2013). TLR7 contributes to the rapid progression but not to the overall fatal outcome of secondary pneumococcal disease following influenza A virus infection. J. Innate Immun..

[97] McNamee L.A., Harmsen A.G. (2006). Both influenza-induced neutrophil dysfunction and neutrophil-independent mechanisms contribute to increased susceptibility to a secondary Streptococcus pneumoniae infection. Infect. Immun..

[98] Small C.L., Shaler C.R., McCormick S., Jeyanathan M., Damjanovic D., Brown E.G., Arck P., Jordana M., Kaushic C., Ashkar A.A. (2010). Influenza infection leads to increased susceptibility to subsequent bacterial superinfection by impairing NK cell responses in the lung. J. Immunol..

[99] Li W., Moltedo B., Moran T.M. (2012). Type I interferon induction during influenza virus infection increases susceptibility to secondary Streptococcus pneumoniae infection by negative regulation of γδ T cells. J. Virol..

[100] Shahangian A., Chow E.K., Tian X., Kang J.R., Ghaffari A., Liu S.Y., Belperio J.A., Cheng G., Deng J.C. (2009). Type I IFNs mediate development of postinfluenza bacterial pneumonia in mice. J. Clin. Investig..

[101] Kash J.C., Walters K.A., Davis A.S., Sandouk A., Schwartzman L.M., Jagger B.W., Chertow D.S., Qi L., Kuestner R.E., Ozinsky A. (2011). Lethal synergism of 2009 pandemic H1N1 influenza virus and Streptococcus pneumoniae coinfection is associated with loss of murine lung repair responses. mBio.

[102] Goulding J., Godlee A., Vekaria S., Hilty M., Snelgrove R., Hussell T. (2011). Lowering the threshold of lung innate immune cell activation alters susceptibility to secondary bacterial superinfection. J. Infect. Dis..

[103] McCullers J.A., McAuley J.L., Browall S., Iverson A.R., Boyd K.L., Normark B.H. (2010). Influenza enhances susceptibility to natural acquisition of and disease due to Streptococcus pneumoniae in ferrets. J. Infect. Dis..

[104] Siegel S.J., Roche A.M., Weiser J.N. (2014). Influenza promotes pneumococcal growth during coinfection by providing host sialylated substrates as a nutrient source. Cell Host Microbe.

[105] Mina M.J., Klugman K.P. (2014). The role of influenza in the severity and transmission of respiratory bacterial disease. Lancet Respir. Med..

[106] Mina M.J., McCullers J.A., Klugman K.P. (2014). Live attenuated influenza vaccine enhances colonization of Streptococcus pneumoniae and Staphylococcus aureus in mice. mBio.

[107] Department of Economic, U.N. (2002). World Population Ageing, 1950–2050.

[108] Goronzy J.J., Weyand C.M. (2013). Understanding immunosenescence to improve responses to vaccines. Nat. Immunol..

[109] Miller R.A. (1996). The aging immune system: Primer and prospectus. Science.

[110] Wick G., Grubeck-Loebenstein B. (1997). The aging immune system: Primary and secondary alterations of immune reactivity in the elderly. Exp. Gerontol..

[111] Shaw A.C., Joshi S., Greenwood H., Panda A., Lord J.M. (2010). Aging of the innate immune system. Curr. Opin. Immunol..

[112] Mahbub S., Brubaker A.L., Kovacs E.J. (2011). Aging of the innate immune system: An update. Curr. Immunol. Rev..

[113] Solana R., Tarazona R., Gayoso I., Lesur O., Dupuis G., Fulop T. (2012). Innate immunosenescence: Effect of aging on cells and receptors of the innate immune system in humans. Semin. Immunol..

[114] Goronzy J.J., Lee W.W., Weyand C.M. (2007). Aging and T-cell diversity. Exp. Gerontol..

[115] Gupta S., Bi R., Su K., Yel L., Chiplunkar S., Gollapudi S. (2004). Characterization of naive, memory and effector CD8+ T cells: Effect of age. Exp. Gerontol..

[116] Vallejo A.N. (2005). CD28 extinction in human T cells: Altered functions and the program of T-cell senescence. Immunol. Rev..

[117] Fulop T., Le Page A., Fortin C., Witkowski J.M., Dupuis G., Larbi A. (2014). Cellular signaling in the aging immune system. Curr. Opin. Immunol..

[118] Ling W., Yan X., Li-Jing Z., Chang T.T., Jie L. (2010). An association between immunosenescence and CD4+ CD25+ regulatory T cells: A systematic review. Biomed. Environ. Sci..

[119] Poland G.A., Kennedy R.B., McKinney B.A., Ovsyannikova I.G., Lambert N.D., Jacobson R.M., Oberg A.L. (2013). Vaccinomics, adversomics, and the immune response network theory: Individualized vaccinology in the 21st century. Semin. Immunol..

[120] Poland G.A., Ovsyannikova I.G., Kennedy R.B., Lambert N.D., Kirkland J.L. (2014). A systems biology approach to the effect of aging, immunosenescence and vaccine response. Curr. Opin. Immunol..

[121] Durando P., Iudici R., Alicino C., Alberti M., de Florentis D., Ansaldi F., Icardi G. (2011). Adjuvants and alternative routes of administration towards the development of the ideal influenza vaccine. Hum. Vaccines.

[122] Monto A.S., Ansaldi F., Aspinall R., McElhaney J.E., Montano L.F., Nichol K.L., Puig-Barberà J., Schmitt J., Stephenson I. (2009). Influenza control in the 21st century: Optimizing protection of older adults. Vaccine.

[123] Hsieh Y.C., Wu T.Z., Liu D.P., Shao P.L., Chang L.Y., Lu C.Y., Lee C.Y., Huang F.Y., Huang L.M. (2006). Influenza pandemics: Past, present and future. J. Formos. Med. Assoc..

[124] Zhang H., Chen L. (2009). Possible origin of current influenza A H1N1 viruses. Lancet Infect. Dis..

[125] Garten R.J., Davis C.T., Russell C.A., Shu B., Lindstrom S., Balish A., Sessions W.M., Xu X., Skepner E., Deyde V. (2009). Antigenic and genetic characteristics of swine-origin 2009 A (H1N1) influenza viruses circulating in humans. Science.

[126] Monto A.S., Black S., Plotkin S.A., Orenstein W.A. (2011). Response to the 2009 pandemic: Effect on influenza control in wealthy and poor countries. Vaccine.

[127] Clegg C.H., Rininger J.A., Baldwin S.L. (2013). Clinical vaccine development for H5N1 influenza. Expert Rev. Vaccines.

[128] Del Giudice G., Weinberger B., Grubeck-Loebenstein B. (2015). Vaccines for the Elderly. Gerontology.

[129] Amorij J.P., Hinrichs W.L., Frijlink H.W., Wilschut J.C., Huckriede A. (2010). Needle-free influenza vaccination. Lancet Infect. Dis..

[130] Neutra M.R., Kozlowski P.A. (2006). Mucosal vaccines: The promise and the challenge. Nat. Rev. Immunol..

[131] Lycke N. (2012). Recent progress in mucosal vaccine development: Potential and limitations. Nat. Rev. Immunol..

[132] Fujihashi K., Sato S., Kiyono H. (2014). Mucosal adjuvants for vaccines to control upper respiratory infections in the elderly. Exp. Gerontol..

[133] Van Ginkel F.W., Jackson R.J., Yuki Y., McGhee J.R. (2000). Cutting edge: The mucosal adjuvant cholera toxin redirects vaccine proteins into olfactory tissues. J. Immunol..

[134] Mutsch M., Zhou W., Rhodes P., Bopp M., Chen R.T., Linder T., Spyr C., Steffen R. (2004). Use of the inactivated intranasal influenza vaccine and the risk of Bell’s palsy in Switzerland. N. Engl. J. Med..

[135] Wegmann F., Gartlan K.H., Harandi A.M., Brinckmann S.A., Coccia M., Hillson W.R., Kok W.L., Cole S., Ho L.P., Lambe T. (2012). Polyethyleneimine is a potent mucosal adjuvant for viral glycoprotein antigens. Nat. Biotechnol..

[136] Stanberry L., Simon J., Johnson C., Robinson P., Morry J., Flack M., Gracon S., Myc A., Hamouda T., Baker J. (2012). Safety and immunogenicity of a novel nanoemulsion mucosal adjuvant W 80 5EC combined with approved seasonal influenza antigens. Vaccine.

[137] Huleatt J.W., Nakaar V., Desai P., Huang Y., Hewitt D., Jacobs A., Tang J., McDonald W., Song L., Evans R.K. (2008). Potent immunogenicity and efficacy of a universal influenza vaccine candidate comprising a recombinant fusion protein linking influenza M2e to the TLR5 ligand flagellin. Vaccine.

[138] Ebrahimi S.M., Tebianian M. (2011). Influenza A viruses: Why focusing on M2e-based universal vaccines. Virus Genes.

[139] Atsmon J., Kate-Ilovitz E., Shaikevich D., Singer Y., Volokhov I., Haim K.Y., Ben-Yedidia T. (2012). Safety and immunogenicity of multimeric-001-a novel universal influenza vaccine. J. Clin. Immunol..

[140] Ben-Yedidia T., Arnon R. (2007). Epitope-based vaccine against influenza. Expert Rev. Vaccines.

[141] Kaur K., Sullivan M., Wilson P.C. (2011). Targeting B cell responses in universal influenza vaccine design. Trends Immunol..

[142] Wei C.J., Boyington J.C., McTamney P.M., Kong W.P., Pearce M.B., Xu L., Andersen H., Rao S., Tumpey T.M., Yang Z.Y. (2010). Induction of broadly neutralizing H1N1 influenza antibodies by vaccination. Science.

[143] Price G.E., Soboleski M.R., Lo C.Y., Misplon J.A., Quirion M.R., Houser K.V., Pearce M.B., Pappas C., Tumpey T.M., Epstein S.L. (2010). Single-dose mucosal immunization with a candidate universal influenza vaccine provides rapid protection from virulent H5N1, H3N2 and H1N1 viruses. PLoS ONE.

[144] Poon L.L., Leung Y.C., Nicholls J.M., Perera P.Y., Lichy J.H., Yamamoto M., Waldmann T.A., Peiris J.M., Perera L.P. (2009). Vaccinia virus-based multivalent H5N1 avian influenza vaccines adjuvanted with IL-15 confer sterile cross-clade protection in mice. J. Immunol..

[145] Suter R., Summerfield A., Thomann-Harwood L.J., McCullough K.C., Tratschin J.D., Ruggli N. (2011). Immunogenic and replicative properties of classical swine fever virus replicon particles modified to induce IFN-α/β and carry foreign genes. Vaccine.

[146] McCullough K.C., Bassi I., Démoulins T., Thomann-Harwood L.J., Ruggli N. (2012). Functional RNA delivery targeted to dendritic cells by synthetic nanoparticles. Ther. Deliv..

[147] Pica N., Palese P. (2013). Toward a universal influenza virus vaccine: Prospects and challenges. Annu. Rev. Med..

[148] Bolton K.J., McCaw J.M., Brown L., Jackson D., Kedzierska K., McVernon J. (2015). Prior Population Immunity Reduces the Expected Impact of CTL-Inducing Vaccines for Pandemic Influenza Control. PLoS ONE.

[149] Baguelin M., Flasche S., Camacho A., Demiris N., Miller E., Edmunds W.J. (2013). Assessing optimal target populations for influenza vaccination programmes: An evidence synthesis and modelling study. PLoS Med..

[150] Pettini E., Prota G., Ciabattini A., Boianelli A., Fiorino F., Pozzi G., Vicino A., Medaglini D. (2013). Vaginal Immunization to Elicit Primary T-Cell Activation and Dissemination. PLoS ONE.

[151] Boianelli A., Pettini E., Prota G., Medaglini D., Vicino A. Identification of a branching process model for adaptive immune response. Proceedings of the 2013 IEEE 52nd Annual Conference on Decision and Control (CDC).

[152] Boianelli A., Pettini E., Prota G., Medaglini D., Vicino A. (2015). A Stochastic Model for CD4+ T Cell Proliferation and Dissemination Network in Primary Immune Response. PLoS ONE.

[153] Meyer-Hermann M., Mohr E., Pelletier N., Zhang Y., Victora G.D., Toellner K.M. (2012). A theory of germinal center B cell selection, division, and exit. Cell Rep..

[154] Victora G.D., Nussenzweig M.C. (2012). Germinal centers. Annu. Rev. Immunol..

[155] Meyer-Hermann M. (2014). Overcoming the Dichotomy of Quantity and Quality in Antibody Responses. J. Immunol..

[156] Chaudhury S., Reifman J., Wallqvist A. (2014). Simulation of B Cell Affinity Maturation Explains Enhanced Antibody Cross-Reactivity Induced by the Polyvalent Malaria Vaccine AMA1. J. Immunol..

[157] Srivastava B., Błażejewska P., Heßmann M., Bruder D., Geffers R., Mauel S., Gruber A.D., Schughart K. (2009). Host genetic background strongly influences the response to influenza a virus infections. PLoS ONE.

[158] Consortium C.C. (2012). The genome architecture of the Collaborative Cross mouse genetic reference population. Genetics.

[159] Bottomly D., Ferris M.T., Aicher L.D., Rosenzweig E., Whitmore A., Aylor D.L., Haagmans B.L., Gralinski L.E., Bradel-Tretheway B.G., Bryan J.T. (2012). Expression quantitative trait Loci for extreme host response to influenza a in pre-collaborative cross mice. G3 Genes Genomes Genet..

[160] Ferris M.T., Aylor D.L., Bottomly D., Whitmore A.C., Aicher L.D., Bell T.A., Bradel-Tretheway B., Bryan J.T., Buus R.J., Gralinski L.E. (2013). Modeling host genetic regulation of influenza pathogenesis in the collaborative cross. PLoS Pathog..

[161] Bailey C.C., Huang I.C., Kam C., Farzan M. (2012). Ifitm3 limits the severity of acute influenza in mice. PLoS Pathog..

[162] Everitt A.R., Clare S., Pertel T., John S.P., Wash R.S., Smith S.E., Chin C.R., Feeley E.M., Sims J.S., Adams D.J. (2012). IFITM3 restricts the morbidity and mortality associated with influenza. Nature.

[163] Xuan Y., Wang L., Li W., Zi H., Guo Y., Yan W., Chen X., Wei P. (2015). IFITM3 rs12252 T > C polymorphism is associated with the risk of severe influenza: A meta-analysis. Epidemiol. Infect..

[164] Mak C.M., Lam C.W., Fong N.C., Siu W.K., Lee H.C.H., Siu T.S., Lai C.K., Law C.Y., Tong S.F., Poon W.T. (2011). Fatal viral infection-associated encephalopathy in two Chinese boys: A genetically determined risk factor of thermolabile carnitine palmitoyltransferase II variants. J. Hum. Genet..

[165] Hatesuer B., Bertram S., Mehnert N., Bahgat M.M., Nelson P.S., Pöhlman S., Schughart K. (2013). TMPRSS2 is essential for influenza H1N1 virus pathogenesis in mice. PLoS Pathog..

[166] Sakai K., Ami Y., Tahara M., Kubota T., Anraku M., Abe M., Nakajima N., Sekizuka T., Shirato K., Suzaki Y. (2014). The host protease TMPRSS2 plays a major role in *in vivo* replication of emerging H7N9 and seasonal influenza viruses. J. Virol..

[167] Tarnow C., Engels G., Arendt A., Schwalm F., Sediri H., Preuss A., Nelson P.S., Garten W., Klenk H.D., Gabriel G. (2014). TMPRSS2 is a host factor that is essential for pneumotropism and pathogenicity of H7N9 influenza A virus in mice. J. Virol..

[168] Kollmus H., Wilk E., Schughart K. (2014). Systems biology and systems genetics-novel innovative approaches to study host—pathogen interactions during influenza infection. Curr. Opin. Virol..

[169] Elowitz M.B., Levine A.J., Siggia E.D., Swain P.S. (2002). Stochastic gene expression in a single cell. Science.

[170] El Samad H., Khammash M., Petzold L., Gillespie D. (2005). Stochastic modelling of gene regulatory networks. Int. J. Robust Nonlinear Control.

[171] Paulsson J. (2004). Summing up the noise in gene networks. Nature.

[172] Eldar A., Elowitz M.B. (2010). Functional roles for noise in genetic circuits. Nature.

